# β-lactam antibiotics induce metabolic perturbations linked to ROS generation leads to bacterial impairment

**DOI:** 10.3389/fmicb.2024.1514825

**Published:** 2024-12-06

**Authors:** Dongyang Ye, Jing Sun, Ran Jiang, Jiashen Chang, Yiming Liu, Xiangzheng Wu, Luqi Li, Yihan Luo, Juan Wang, Kangkang Guo, Zengqi Yang

**Affiliations:** ^1^College of Veterinary Medicine, Northwest A&F University, Yangling, Shaanxi, China; ^2^Key Laboratory of Ruminant Disease Prevention and Control (West), Ministry of Agriculture and Rural Affairs, Yangling, Shaanxi, China; ^3^Key Laboratory of Animal-Derived Bacterial Resistance Monitoring (Co-Construction), Ministry of Agriculture and Rural Affairs, Yangling, Shaanxi, China; ^4^Experimental Animal Center, Northwest A&F University, Yangling, Shaanxi, China; ^5^Life Science Research Core Services, Northwest A&F University, Yangling, Shaanxi, China

**Keywords:** β-lactam, antibiotics, metabolomics, metabolic perturbations, *E. coli*

## Abstract

Understanding the impact of antibiotics on bacterial metabolism is crucial for elucidating their mechanisms of action and developing more effective therapeutic strategies. β-lactam antibiotics, distinguished by their distinctive β-lactam ring structure, are widely used as antimicrobial agents. This study investigates the global metabolic alterations induced by three β-lactam antibiotics-meropenem (a carbapenem), ampicillin (a penicillin), and ceftazidime (a cephalosporin)-in *Escherichia coli*. Our comprehensive metabolic profiling revealed significant perturbations in bacterial metabolism, particularly in pathways such as glutathione metabolism, pantothenate and CoA biosynthesis, pyrimidine metabolism, and purine metabolism. Antibiotic treatment markedly increased reactive oxygen species levels, with meropenem reaching nearly 200 ± 7%, ampicillin at 174 ± 11%, and ceftazidime at 152 ± 7%. Additionally, β-lactam antibiotics elevated 8-OHdG levels to 4.73 ± 0.56-fold for meropenem, 2.49 ± 0.19-fold for ampicillin, and 3.19 ± 0.34-fold for ceftazidime; 8-OHG levels increased to 5.57 ± 0.72-fold for meropenem, 3.08 ± 0.31-fold for ampicillin, and 4.45 ± 0.66-fold for ceftazidime, indicating that oxidative stress enhances oxidative damage to bacterial DNA and RNA. Notably, we observed a selective upregulation of specific amino acids associated with cellular repair mechanisms, indicating a metabolic adaptation to counteract oxidative damage. These findings illustrate that β-lactam antibiotics induce a complex metabolic perturbations associated with ROS production, potentially compromising critical cellular components. This study enhances our understanding of the intricate relationship between antibiotic action and bacterial metabolism, providing valuable insights for developing effective strategies against antibiotic-resistant pathogens.

## 1 Introduction

The continuous discovery and development of novel antibiotics have led to a gradual deepening of researchers’ understanding regarding their mechanisms of action. The traditional research paradigm has primarily focused on analyzing the interaction between antibiotics and direct targets within bacterial cells (Kohanski et al., 2010; Nonejuie et al., 2013; Theuretzbacher et al., 2023). This target-centric research approach has provided valuable insights into the precise disruption of bacterial cell functions by antibiotics (Sodhi et al., 2023), an excessive reliance on this singular perspective also imposes certain limitations. While the majority of current systems biology studies in antibiotics primarily focus on unraveling the intricacies of transcriptional regulatory networks and biofilms (Shree et al., 2023), mounting evidence suggests that alterations in cellular metabolic states also play a pivotal role in governing antibiotic sensitivity (Lopatkin et al., 2021). Understanding metabolic perturbations in antibiotic resistance provides crucial insights into bacterial adaptations of their metabolic networks to survive and persist under antibiotic stress, potentially unveiling novel therapeutic targets. By elucidating these metabolic adaptations, researchers can devise more efficacious strategies to combat bacterial resistance, such as designing combination therapies that disrupt metabolic survival mechanisms and augment the effectiveness of existing antibiotics.

Bacterial survival and proliferation heavily rely on their metabolic activities, which not only support fundamental physiological processes but also influence the cell’s capacity to adapt to its external environment (Goossens et al., 2020). For instance, defects in the TCA cycle can profoundly impact bacterial stress response and survival strategies when confronted with antibiotics. The inhibition of the TCA cycle may prompt bacteria to modulate their metabolic pathways and rely on alternative metabolites for sustenance, resulting in distinct antibiotic resistance phenotypes (Stokes et al., 2019). Moreover, perturbation of the TCA cycle can disrupt bacterial energy homeostasis, leading to alterations in antibiotic targets or enhanced utilization of alternative metabolic pathways that significantly bolster bacterial viability (Yao et al., 2024). Thoroughly investigating the metabolic changes induced by antibiotics and their impact on bacterial cell survival is imperative for current endeavors to enhance our antibiotic arsenal (Ye et al., 2022, 2024).

β-lactam antibiotics, distinguished by their unique β-lactam ring structure, serve as widely utilized antimicrobial agents. These antibiotics function by binding to PBPs in bacteria, which disrupts cell wall synthesis and leads to bacterial cell lysis and death (Arad et al., 2023; Lewis et al., 2024). To identify the global changes to bacterial metabolism following β-lactam antibiotics treatment, we profiled metabolic alterations in *E. coli* resulting from treatment with three different β-lactam antibiotics: meropenem (MEM, a carbapenem), ampicillin (AMP, a penicillin), ceftazidime (CAZ, a cephalosporin). Our observations indicate that these antibiotic classes produce both common and unique metabolic responses, with all treatment conditions leading to a significant decrease in essential energy metabolites. A reduction in antioxidant metabolites further suggests that the oxidative stress triggered by antibiotics may surpass the capacity of bacterial antioxidant defenses. Notably, specific amino acids linked to cellular repair exhibited selective upregulation, reflecting a metabolic adaptation to address oxidative damage. Our findings demonstrate that β-lactam antibiotics initiate a complex array of metabolic perturbations in bacteria that are correlated with the production of ROS, capable of damaging critical cellular components. This study advances our understanding of the intricate relationship between antibiotic action and bacterial metabolism, providing valuable insights for developing effective strategies against antibiotic-resistant pathogens.

## 2 Materials and methods

### 2.1 Bacterial strains and reagents

*E. coli* ATCC 25922 was used for the studies. Bacterial strains were cultivated under the conditions of Luria–Bertani (LB) medium at a temperature of 37°C. MEM, AMP, CAZ and ascorbic acid were purchased from Solarbio (Beijing). The ROS Detection Assay Kit was purchased from GENMED Scientifics Inc., USA. Methanol, acetonitrile, formic acid, and ethanol were sourced from Fisher Chemical, USA.

### 2.2 SEM analysis

Bacterial cultures of *E. coli* ATCC 25922 were prepared and stimulated with MEM (0.5 μg/mL), AMP (3.5 μg/mL), and CAZ (1.5 μg/mL) at an OD_600_ of approximately 0.30. The cells were then harvested through centrifugation, washed with PBS, and subsequently resuspended in a 3% glutaraldehyde solution for fixation purposes. After fixation for a duration of 1–2 h in a 1% osmium tetroxide solution, the samples underwent three consecutive washes with ultrapure water lasting for 10 min each. Dehydration was conducted using a sequential series of ethanol solutions: 30, 50, 70, 90, and three repetitions of 100% concentration, with each step lasting for a duration of 15 min. Subsequently, the samples were deposited onto silicon wafers via pipetting and affixed to a sample stage using conductive adhesive. The mounted samples were then coated with gold through ion sputtering utilizing an advanced sputtering instrument. Imaging analysis was performed employing the JSM-IT700HR scanning electron microscope (JEOL, Japan). Initially, low magnification observations were made to evaluate the overall morphology of each sample, followed by high-resolution imaging targeting specific regions in order to identify distinct bacterial morphological changes.

### 2.3 Untargeted metabolomics analysis

Bacterial cultures of *E. coli* ATCC 25922 were prepared and stimulated with MEM (0.5 μg/mL), AMP (3.5 μg/mL), CAZ (1.5 μg/mL) at an OD_600_ of approximately 0.30 for 1 h. The cultures were quenched using a methanol/ethylene glycol mixture at −60°C and subsequently washed with a 0.85% NaCl solution. Following this, cell pellets were extracted with boiling ethanol/water (75:25, v/v) at 95°C. Metabolite separation was executed through both reversed-phase (RP) and hydrophilic interaction liquid chromatography (HILIC), with detection performed using a UHPLC-TOF/MS system (AB Sciex, 6600 + , Framingham, USA) in both positive and negative electrospray ionization modes. For RP separation, a Waters BEH Shield RP C_18_ column (2.1 mm × 100 mm, 1.7 μm) was utilized, employing mobile phases consisting of solvent A (0.1% formic acid in water) and solvent B (0.1% formic acid in acetonitrile) for ESI^+^ mode, and solvent A (5 mM ammonium acetate in water) with solvent B (5 mM ammonium acetate in acetonitrile) for ESI^–^ mode. The gradient for this separation was as follows: from 0 to 1 min, 2% B; from 2 to 10 min, 2–40% B; from 10 to 11 min, 40–98% B; from 11 to 12 min, maintaining at 98% B; from 12 to 12.1 min, returning from 98% to 2% B; and from 12.1 to 15 min, at 2% B. HILIC separation was performed using a Waters BEH Amide column (100 mm × 2.1 mm, 1.7 μm). In this case, solvent A consisted of 0.1% formic acid plus 5 mM ammonium acetate in water, while solvent B was 0.1% formic acid in acetonitrile for ESI^+^ mode, and solvent A (5 mM ammonium acetate in water) with solvent B (5 mM ammonium acetate in acetonitrile) for ESI^–^ mode. The linear gradient program for HILIC was as follows: from 0 to 2 min, 95% B; from 2 to 8 min, decreasing from 95 to 70% B; from 8 to 9 min, 70 to 50% B; from 9 to 10 min, maintaining at 50% B; from 10 to 10.1 min, increasing from 50% to 95% B; and from 10.1 to 15 min, remaining at 95% B. The flow rate was set at 0.3 mL/min with an injection volume of 2 μL. The triple-TOF/MS parameters were configured as follows: curtain gas at 30 psi; ion source at ±4500 V; ion spray probe temperature at 500°C; nebulizer gas (N2) at 50 psi; auxiliary heating gas (N_2_) at 50 psi; and a declustering potential of 80 eV. Stepped collision energies were set to 20, 35, and 50 eV, with a mass range of 50–1200 Da, operating in independent data acquisition mode.

The metabolomic samples were analyzed using UHPLC-Triple TOF-MS/MS (AB Sciex) detection to acquire raw data acquisition files. Subsequently, the raw data files (.wiff) were imported into Progenesis QI software for preprocessing, which encompassed chromatographic peak extraction, retention time calibration, peak identification, noise filtering, and peak matching and data normalize. This preprocessing step generated a comprehensive data matrix containing crucial information such as mass-to-charge ratio (m/z), retention time (RT), elemental composition, %RSD, and peak abundance. For multivariate statistical analysis, EZinfo software was employed to perform principal component analysis (PCA). PCA effectively reduced dimensionality while minimizing potential issues like feature reduction, noise redundancy or overfitting by representing each sample with minimal data points. Consequently, this approach enabled high-variance dimensions or features to accurately represent the sample clusters or outliers in metabolic phenotypes. The Orthogonal Projection to Latent Structures-Discriminant Analysis (OPLS-DA) aims to minimize the sum of squared errors in order to identify the optimal function that best matches the data and discerns the key differentiating variables. In OPLS-DA analysis, outliers in S-pot plots, which deviate significantly from the central position, are filtered based on Variable Importance in Projection (VIP) values exceeding 1.0. Detected features meeting specific criteria including *p* < 0.05, %RSD < 20%, VIP > 1, and fold change > 1.5 are selected for identifying potential differential metabolites. Metabolite identification was accomplished through database searches against the ECMDB.^1^

### 2.4 Determination of intracellular ROS accumulation

Individual colonies of *E. coli* ATCC 25922 were carefully isolated and subsequently inoculated into sterile LB broth for overnight culture at 37°. The overnight cultures were then transferred to conical flasks containing fresh LB broth and grown until the optical density (OD_600_) reached approximately 0.30. Following this, the cultures were centrifuged at 8,000 rpm for 5 min and washed twice with 30 mL of sterile saline solution. The resulting pellet was resuspended in M9 minimal medium enriched with ammonium acetate (10 mM), MgSO_4_ (1 mM), and CaCl_2_ (100 μM). To these resuspended cultures, meropenem (MEM) at 0.5 μg/mL, ampicillin (AMP) at 3.5 μg/mL, and cephazolin (CAZ) at 1.5 μg/mL were added, along with 10 mM hydrogen peroxide (H_2_O_2_) as the positive control. Each treatment group was set up in triplicate and incubated at 37° for 1 h prior to the assessment of intracellular reactive oxygen species (ROS) levels using a High-Quality Fluorescent Assay Kit for ROS (Genmed Scientific Inc., USA).

### 2.5 Inhibition of intracellular ROS

*E.coli* ATCC 25922 was adjusted to achieve a turbidity corresponding to a McFarland standard of 0.5 and subsequently diluted in 10 mL of M9 media at a 1:100 ratio, resulting in an estimated concentration of 10^6^ CFU/mL. To the diluted cultures, meropenem (MEM) was added at a concentration of 0.5 μg/mL, ampicillin (AMP) at 3.5 μg/mL, and cephazolin (CAZ) at 1.5 μg/mL, with 10 mM hydrogen peroxide (H_2_O_2_) serving as the positive control. Furthermore, ascorbic acid was included in the antibiotic treatment group as a ROS inhibitor at a concentration of 10 mM. ROS levels were measured after an incubation period of 1 h.

### 2.6 ROS-induced nucleic acid damage

Overnight cultures were diluted at a ratio of 1: 250 in 25 mL of LB medium contained within 250 mL baffled flasks and incubated until an OD_600_ of 0.30 was reached. Subsequently, the cells underwent treatment with MEM (0.5 μg/mL), AMP (3.5 μg/mL), CAZ (1.5 μg/mL), and H_2_O_2_ (10 mM) for each specific group for a duration of 1 h. Following treatment, the cells were harvested and subjected to centrifugation at 4000 rpm for 10 min using a benchtop swinging-bucket centrifuge. After washing with PBS, the resulting pellets were resuspended in 400 μL of 1% SDS dissolved in dH_2_O. The resuspended samples were transferred into Lysing Matrix B tubes (MPBio) and vortexed three times for 45 s each, allowing the samples to cool on ice between vortexing periods. RNA extraction was performed utilizing a phenol-chloroform method, while DNA purification was achieved with the QIAmp DNA Mini kit. The quantification of 8-OHdG was carried out using the OxiSelect Oxidative DNA Damage ELISA kit (Cell Biolabs), and the assessment of 8-OHG levels was performed with the OxiSelect Oxidative RNA Damage ELISA kit (Cell Biolabs). Each sample underwent analysis in six replicates.

### 2.7 Statistical analysis

Statistical analysis was conducted using unpaired two-sided Student’s *t*-tests or one-way ANOVA to evaluate significance. The significance levels were defined as follows: *P < 0.05, **P < 0.01.

## 3 Results

### 3.1 Selection of concentrations that perturb bacterial metabolism via β-lactam antibiotics

**FIGURE 1:**
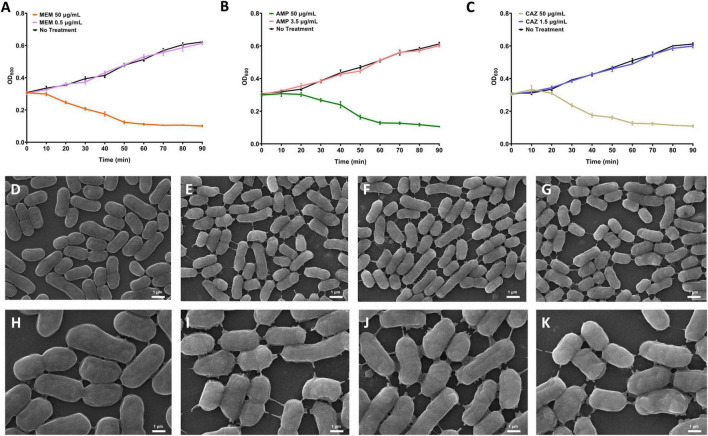
Selection of concentrations that perturb bacterial metabolism via β-lactam antibiotics. **(A–C)** The growth curve of *E. coli* stimulated by the presence of MEM, AMP, and CAZ. **(D)** Control group, 10000×. **(E)** MEM stimulates *E. coli*, 10000×. **(F)** AMP stimulates *E. coli*, 10000×. **(G)** CAZ stimulates *E. coli*, 10000×. **(H)** control group, 20000×. **(I)** MEM stimulates *E. coli*, 20000×. **(J)** AMP stimulates *E. coli*, 20000×. **(K)** CAZ stimulates *E. coli*, 20000×.

β-lactam antibiotics can induce bacterial cell lysis and subsequent death, necessitating the establishment of non-lytic concentrations to investigate metabolic disturbances caused by these antibiotics. Leakage of intracellular metabolites due to cell lysis may lead to data loss and potentially inaccurate experimental outcomes. In this study, we investigated the effects of three distinct β-lactam antibiotics, namely MEM, AMP, and CAZ, on *E. coli* ATCC 25922. Upon reaching an OD_600_ of approximately 0.3, the antibiotics were introduced, and the changes in OD_600_ values were monitored over time to determine non-lytic concentrations for subsequent experiments. Through systematic screening, we identified that growth of *E. coli* ATCC 25922 remained unaffected at concentrations of 0.5 μg/mL for MEM, 3.5 μg/mL for AMP, and 1.5 μg/mL for CAZ (Figures 1A–C). These concentrations were meticulously selected to strike a delicate balance: low enough to prevent cell lysis and minimize confounding metabolic impacts, yet sufficiently high to induce detectable stress responses in the bacterial population. Subsequent examination using scanning electron microscopy (SEM) revealed no significant alterations in cell morphology or membrane structure of *E.coli* ATCC 25922 at these specific antibiotic concentrations (Figures 1D–K). These findings enabled us to establish appropriate antibiotic dosages for subsequent experiments, thus ensuring methodological clarity and reliability in investigating the mechanisms underlying antimicrobial agent-mediated disruption of bacterial metabolism.

### 3.2 Differential metabolic profile mediated by β-lactam antibiotics

To characterize the impact of different classes of β-lactam antibiotics on bacterial metabolism, we employed three bactericidal agents MEM, AMP, and CAZ, to stimulate *E. coli* and investigate its metabolic perturbations following treatment with distinct categories of antibacterial drugs (carbapenems, penicillins, and cephalosporins). Utilizing ultra-high performance liquid chromatography-quadrupole time-of-flight mass spectrometry (UHPLC-Q-TOF/MS) technology, we identified alterations in intracellular metabolites of bacteria. To further elucidate the specific effects exerted by each category of antibacterial drug on *E. coli* metabolism, hierarchical clustering analysis was performed on differential metabolites derived from *E. coli* treated with these three different types of β-lactam antibiotics. The heatmaps illustrate the differential metabolites between antibiotic-treated groups and control groups as well as their hierarchical clustering results; wherein each column represents a specific sample while each row corresponds to a distinct metabolite. The dendrogram positioned atop the heatmaps reveal the clustering relationship among samples, indicating similarities in bacterial metabolic patterns under specific treatment conditions. To improve the interpretability of the data, each metabolite’s functional relevance could be annotated directly on the heatmaps, and bar charts could include expanded legends describing each group’s significance, thereby enhancing the comprehensive understanding of the metabolomic changes induced by antibiotics treatment.

**FIGURE 2:**
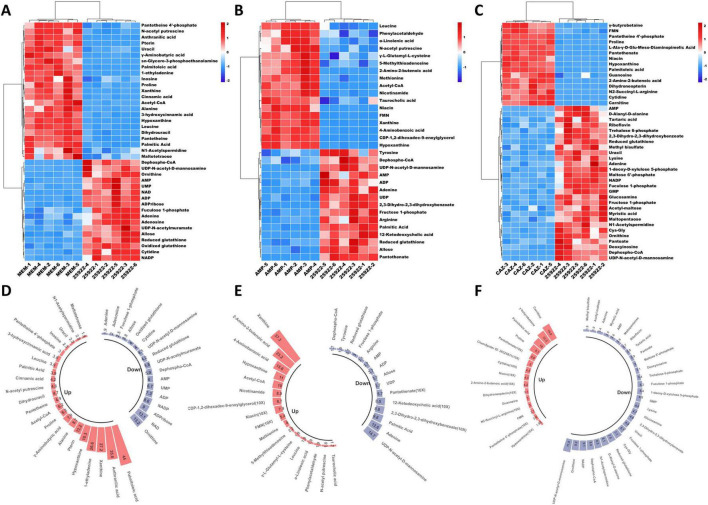
Differential metabolic profile mediated by β-lactam antibiotics. **(A)** Differential metabolites after stimulation of *E. coli* by MEM. **(B)** Differential metabolites after stimulation of *E. coli* by AMP. **(C)** Differential metabolites after stimulation of *E. coli* by CAZ. **(D)** The alteration in metabolite levels following MEM stimulation of *E. coli*. **(E)** The alteration in metabolite levels following AMP stimulation of *E. coli*. **(F)** The alteration in metabolite levels following CAZ stimulation of *E. coli*.

We conducted a metabolomic assessment utilizing heatmap clustering analysis to evaluate the impact of MEM treatment on *E. coli* relative to a control group. The results clearly illustrate that MEM treatment has a substantial effect on the *E. coli* metabolome (Figure 2A). Among the 12 samples analyzed, which included both MEM-treated and control groups, we identified a total of 40 differential metabolites, with 23 showing increased levels and 17 exhibiting decreased levels. The upregulated metabolites span various metabolic categories, including coenzymes such as pantheine 4′-phosphate, pantetheine, and acetyl-CoA. Polyamine metabolism was reflected in the increased abundance of compounds like N-acetyl putrescine and N1-acetylspermidine. We also observed enhancements in metabolites linked to amino acid metabolism, specifically γ-aminobutyric acid, proline, alanine, and leucine. Metabolites associated with phenylalanine metabolism, including cinnamic acid and 3-hydroxycinnamic acid, as well as those related to fatty acid metabolism, such as palmitoleic acid, palmitic acid, and sn-glycero-3-phosphoethanolamine, were also upregulated. Furthermore, nucleotide metabolism showed increased levels of anthranilic acid, pterin, 1-ethyladenine, inosine, xanthine, hypoxanthine, dihydrouracil, and uracil, alongside carbohydrate metabolism indicators like maltotetraose. The downregulated metabolites were primarily associated with energy metabolism, including dephospho-CoA, AMP, ADP, oxidized glutathione, and NADP. Additionally, we noted a decline in metabolites involved in cell structural synthesis, such as UDP-N-acetylmuramate, a vital component of the cell wall, UDP-N-acetyl-D-mannosamine, integral to the cell membrane, and fuculose 1-phosphate. The antioxidant and stress response pathways were also affected, with reductions in both reduced and oxidized glutathione. Other downregulated metabolites included those related to sugar metabolism, such as allose, as well as nucleotide and nucleic acid metabolism, represented by UMP, ADPribose, adenosine, and cytidine. Lastly, urea metabolism was reflected by a decrease in ornithine (Figure 2D).

We also conducted a metabolomic analysis on *E. coli* treated with AMP, which revealed significant changes in the concentrations of 32 metabolites, comprising 17 upregulated and 15 downregulated compounds (Figure 2B). Among the upregulated metabolites, we identified essential cofactors and coenzymes, such as acetyl-CoA, FMN, nicotinamide, and niacin. Additionally, metabolites related to amino acid metabolism and biosynthesis, including leucine, methionine, γ-L-glutamyl-L-cysteine, and 2-amino-2-butenenoic acid, showed increased levels. Notably, polyamine metabolism was reflected in the upregulation of N-acetyl putrescine, while lipid metabolism exhibited enhancements in taurocholic acid, α-linolenic acid, and CDP-1,2-dihexadec-9-enoylglycerol. Moreover, compounds associated with nucleotide metabolism, such as 5-methylthioadenosine, xanthine, and hypoxanthine, were also elevated, alongside phenylalanine metabolism intermediates like phenylacetaldehyde and folate metabolism-related substances including 4-aminobenzoic acid. Conversely, downregulated metabolites were primarily linked to energy metabolism, exemplified by reductions in dephospho-CoA, AMP, and ADP levels. Additionally, compounds integral to cell structure synthesis, including the cell wall component UDP-N-acetyl-D-mannosamine and the cell membrane component palmitic acid, were notably decreased. The analysis also highlighted downregulation in antioxidant and stress response pathways, such as reduced glutathione, along with changes in phenylalanine metabolism (e.g., 2,3-dihydro-2,3-dihydroxybenzoate), signaling pathways (e.g., tyrosine and arginine), carbohydrate metabolism (e.g., fructose 1-phosphate and allose), and nucleotide and nucleic acid metabolism (e.g., adenine and UDP). Also, we observed the reduced activity of certain lipid metabolism pathways, including the downregulation of 12-ketodeoxycholic acid, as well as specific cofactors like pantothenate (Figure 2E).

Metabolomic analysis of *E. coli* subjected to treatment with CAZ revealed significant alterations in the concentrations of 43 metabolites, with 14 metabolites upregulated and 29 downregulated (Figure 2C). The upregulated metabolites predominantly comprised coenzymes and cofactors, including FMN, pantetheine 4′-phosphate, pantothenate, and niacin. Additionally, we observed increases in metabolites related to amino acid metabolism and biosynthesis, such as proline, L-Ala-γ-D-glu-meso-diaminopimelic acid, 2-amino-2-butenonic acid, and N2-succinyl-L-arginine. Compounds associated with nucleotide metabolism, including hypoxanthine, guanosine, and cytidine, were also elevated, along with lipid metabolism-related metabolites like palmitoleic acid, choline metabolism compounds such as γ-butyrobetaine, and folate metabolism-related substances including dihydroneopterin. The downregulated metabolites were primarily involved in energy metabolism (e.g., AMP, NADP) and included coenzymes or cofactors such as desphospho-CoA and pantoate. Additionally, metabolites associated with carbohydrate metabolism, like trehalose 6-phosphate, maltose 6′-phosphate, fructose 1-phosphate, acetyl-maltose, and maltopentaose, exhibited reduced levels. Lipid metabolism and cell membrane synthesis were also affected, as evidenced by decreased concentrations of metabolites like myristic acid, fucose 1-phosphate, carnitine, and glucosamine. Furthermore, metabolites relevant to cell wall synthesis, such as D-alanyl-D-alanine and UDP-N-acetyl-D-mannosamine, were downregulated, alongside antioxidant compounds and those involved in stress response, including reduced glutathione and tartaric acid. CAZ treatment induced a series of metabolic shifts in *E. coli*, characterized by diminished energy metabolism, inhibition of cell membrane and wall synthesis, and a weakened stress response and antioxidant capacity. In response to this challenge, the bacteria selectively upregulated specific metabolites associated with cellular repair, energy metabolism, and stress response, while simultaneously downregulating metabolites linked to cell growth and division (Figure 2F). Detailed results can be found in the Supplementary Tables 1–3.

### 3.3 Reprogramming of bacterial metabolic pathways induced by β-lactam antibiotics

**FIGURE 3:**
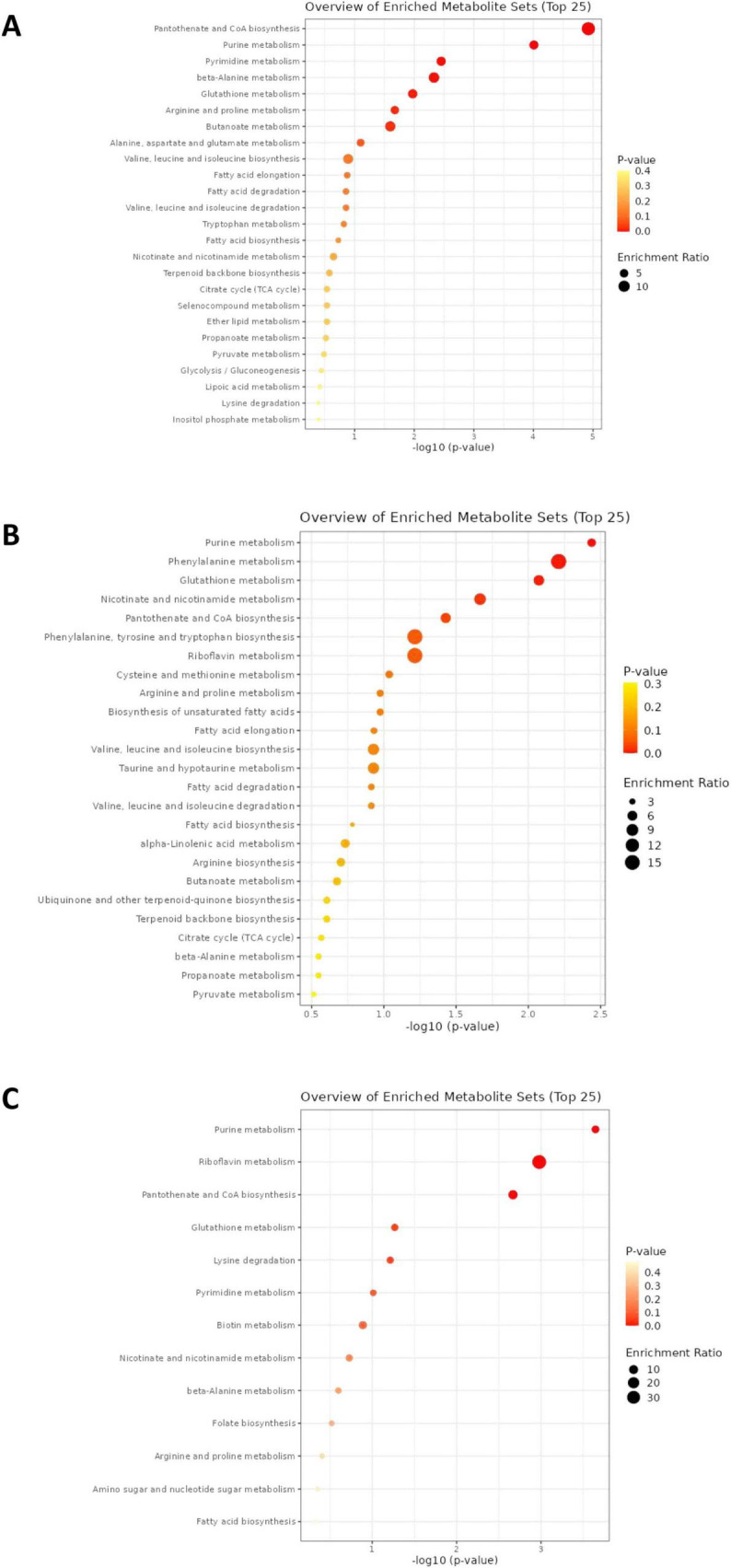
Enrichment analysis of differential metabolites. **(A)** Enrichment analysis of differential metabolites in *E. coli* stimulated by MEM. **(B)** Enrichment analysis of differential metabolites in *E. coli* stimulated by AMP. **(C)** Enrichment analysis of differential metabolites in *E. coli* stimulated by CAZ.

To further elucidate the mechanisms underlying the metabolic alterations in *E. coli* subjected to different β-lactam antibiotic treatments, we conducted KEGG pathway enrichment analysis on the identified differential metabolites and visualized the results. Figures 3A–C illustrates the statistically significant enriched metabolic pathways for each treatment group, with the x-axis representing the -log (*P*-value) and the y-axis indicating the various metabolic pathways. The red dots signify pathways with higher significance, whereas the yellow dots represent those with lower significance.

In the MEM treatment group, pathways such as pantothenate and CoA biosynthesis, β-alanine metabolism, and butanoate metabolism were significantly enriched (Figure 3A). These findings suggest that under MEM-induced stress, *E. coli* actively engages these metabolic pathways, which may play a crucial role in cellular regulation and the adjustment of energy metabolism. For the AMP treatment group, significant enrichment was observed in pathways related to riboflavin metabolism, phenylalanine metabolism, and the biosynthesis of phenylalanine, tyrosine, and tryptophan (Figure 3B). This indicates that *E. coli* may enhance the metabolism of related cofactors and aromatic amino acids in response to the challenges posed by AMP. In the CAZ treatment group, pathways such as pantothenate and CoA biosynthesis, glutathione metabolism, and riboflavin metabolism were similarly enriched (Figure 3C). This suggests that *E. coli* attempts to modulate these vital metabolic pathways to cope with the antibiotic pressure exerted by CAZ, thereby preserving normal cellular physiological functions. The distinct patterns of metabolic regulation observed in *E. coli* under different antibiotic treatments highlight the specific adaptive mechanisms employed by the bacteria.

### 3.4 Upregulation and downregulation of metabolites in key altered pathways

**FIGURE 4:**
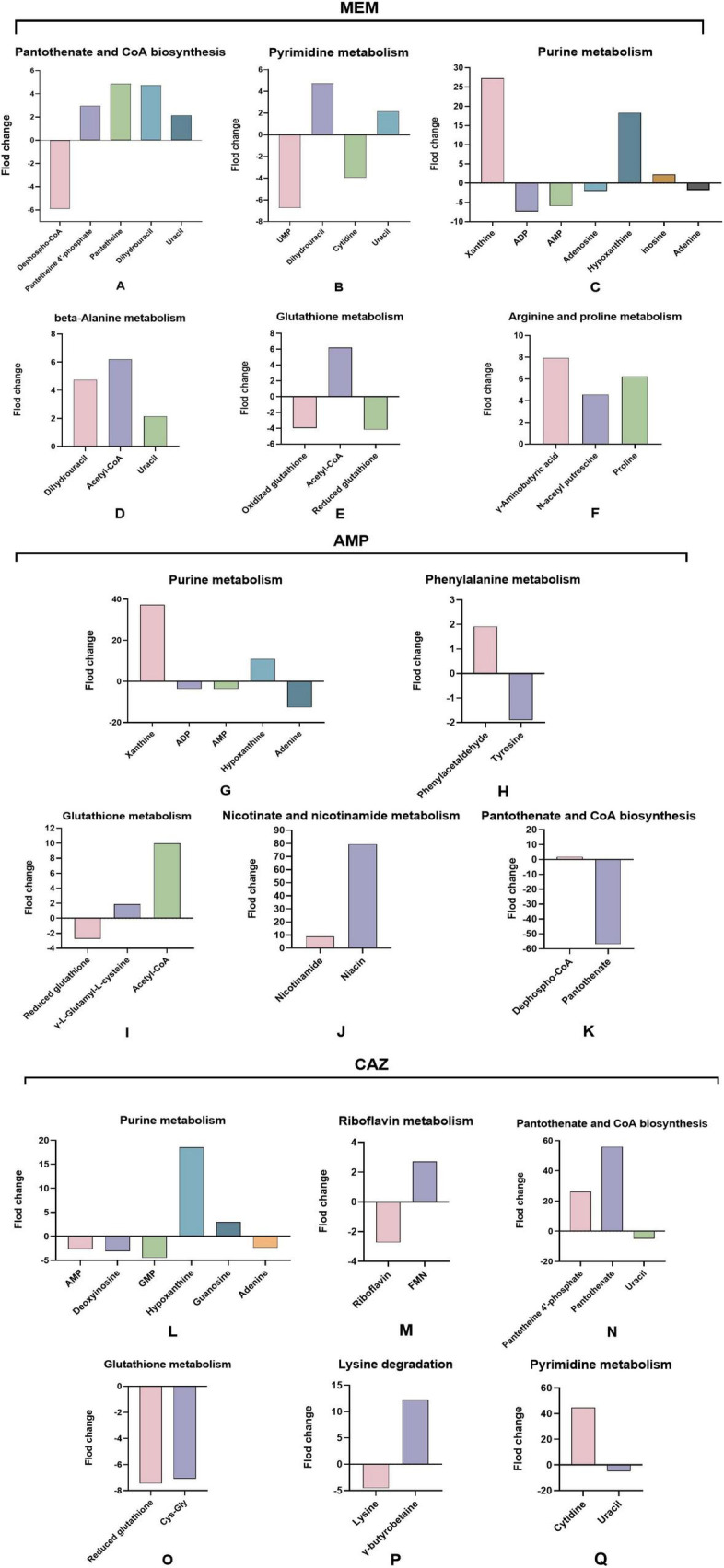
Upregulation and downregulation of metabolites in key altered pathways. **(A–F)** Alterations in metabolites within key disrupted metabolic pathways of *E. coli* following MEM stimulation. **(G–K)** Alterations in metabolites within key disrupted metabolic pathways of *E. coli* following AMP stimulation. **(L–Q)** Alterations in metabolites within key disrupted metabolic pathways of *E. coli* following CAZ stimulation.

To further investigate the metabolic alterations associated with *E. coli* under varying antibiotic treatments, we conducted a comprehensive analysis of relative concentration changes in key metabolites across multiple metabolic pathways. The bar graphs depict the effects of MEM treatment on specific metabolites. In the pantothenate and CoA biosynthesis pathway, we observed a significant downregulation of dephosphorylated-CoA. Conversely, metabolites such pantetheine 4′-phosphate, pantetheine, and dihydrouracil showed upward trends, with uracil also experiencing an increase (Figure 4A). Within the pyrimidine metabolism pathway, concentrations of UMP and cytidine decreased, while levels of dihydrouracil and uracil increased (Figure 4B). In the purine metabolism pathway, there were notable increases in xanthine and hypoxanthine concentrations, which rose by 26.6-fold and 18.3-fold, respectively, while ADP and AMP levels were downregulated, with slight decreases observed in adenosine and adenine (Figure 4C). Notable upregulation of dihydropyrimidine, acetyl-CoA, and uracil was observed in the beta-alanine metabolism pathway (Figure 4D). In glutathione metabolism, both oxidized and reduced glutathione concentrations were significantly downregulated, while acetyl-CoA levels increased (Figure 4E). Furthermore, significant elevations in γ-aminobutyric acid, N-acetyl-L-proline, and proline concentrations were detected in the arginine and proline metabolism pathway (Figure 4F).

Following treatment with AMP, we observed a substantial increase in xanthine concentrations in the purine metabolism pathway, with a striking rise of 37.3-fold. Hypoxanthine levels also increased; however, ADP and AMP levels were downregulated, and adenine exhibited a significant decline of 12.6-fold (Figure 4G). In phenylalanine metabolism, phenylacetaldehyde showed slight upregulation, while tyrosine concentrations decreased slightly (Figure 4H). Within glutathione metabolism, reduced glutathione levels diminished, accompanied by a modest increase in γ-L-glutamyl-L-cysteine, and an overall rise in acetyl-CoA concentrations was noted (Figure 4I). The nicotinate and nicotinamide metabolism pathway revealed a dramatic increase in niacin levels, rising 79.4-fold, contrasted by a more modest increase of 8.9-fold in nicotinamide (Figure 4J). In the pantothenate and CoA biosynthesis pathway, dephospho-CoA showed a slight increase, whereas pantothenate concentration fell sharply by 57.0-fold (Figure 4K).

After treatment with CAZ, we observed a slight downregulation of AMP, deoxyadenosine, and GMP within purine metabolism, accompanied by a marked 18.5-fold increase in hypoxanthine levels and a modest rise in guanosine (Figure 4L). In riboflavin metabolism, while FMN levels were slightly downregulated, riboflavin itself showed a modest increase (Figure 4M). The pantothenate and CoA biosynthesis pathway exhibited significant increases in both pantetheine 4′-phosphate and pantothenate, whereas uracil levels experienced a slight decline (Figure 4N). In glutathione metabolism, reduced glutathione and Cys-Gly were significantly downregulated (Figure 4O). Additionally, in the context of lysine degradation, we noted a decrease in lysine levels coinciding with an elevation in γ-butyrobetaine (Figure 4P). Within pyrimidine metabolism, cytidine levels significantly increased, while uracil showed a slight decrease (Figure 4Q).

### 3.5 Intracellular accumulation of ROS in bacteria induced by β-lactam antibiotics

Oxidative stress serves as a critical indicator of cellular dysfunction, often evaluated through the reduced glutathione (GSH) to oxidized glutathione (GSSG) ratio. A decline in this ratio is widely recognized as a hallmark of oxidative damage within cells (Belenky et al., 2015). In the present study, we investigated the metabolic disruptions induced by three commonly utilized β-lactam antibiotics, namely MEM, AMP, and CAZ, in *E. coli*. Treatment with these antibiotics resulted in a significant reduction in the GSH/GSSG ratio, indicating that exposure to MEM, AMP, and CAZ triggered oxidative stress in *E. coli*.

**FIGURE 5:**
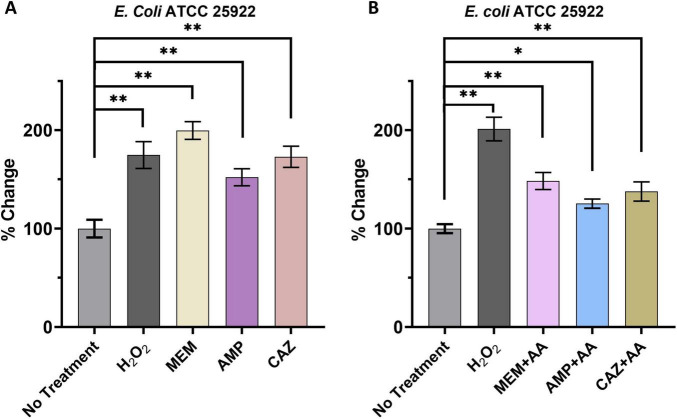
Intracellular accumulation of ROS in bacteria induced by β-lactam antibiotics. **(A)** ROS accumulation dynamics upon antimicrobial stimulation. **(B)** Repercussions of ROS inhibitors on intracellular ROS levels. *p < 0.05, **p < 0.01.

To validate these observations, we measured intracellular ROS levels in *E. coli* post-antibiotic treatment using a ROS detection kit. The findings demonstrated a significant increase in ROS levels across all three antibiotics, as well as in the positive control, hydrogen peroxide. Notably, MEM produced the most substantial elevation in ROS, reaching nearly 200 ± 7% of baseline levels. In comparison, AMP and CAZ led to increases of 174 ± 11 and 152 ± 7%, respectively, while the hydrogen peroxide treatment group recorded 172 ± 8% (Figure 5A). These results provide robust evidence that antibiotic treatment significantly raises ROS levels within *E. coli*. The differential ROS generation observed among the antibiotics may reflect variations in how each antibiotic disrupts bacterial membrane integrity or interferes with metabolic pathways, necessitating further studies to delineate these mechanisms. We also observed varying levels of intracellular accumulation of ROS induced by three different types of carbapenem antibiotics, with MEM exhibiting the highest level. This disparity may be attributed to meropenem’s capacity to disrupt cell wall integrity, potentially triggering more mitochondrial and electron transport chain abnormalities within bacteria, consequently leading to increased electron leakage and a substantial elevation in ROS production.

To further investigate the connection between elevated ROS levels and the action of antibiotics, we conducted experiments to inhibit ROS production using ascorbic acid (AA), a known antioxidant, alongside each antibiotic. The introduction of AA significantly mitigated the increase in ROS levels induced by all three antibiotics. Specifically, ROS levels in the MEM, AMP, and CAZ treatment groups decreased to 201 ± 9, 148 ± 7, and 125 ± 3%, respectively (Figure 5B). These findings reinforce the conclusion that the administration of various β-lactam antibiotics effectively elevates ROS levels in bacterial cells, an effect that can be reversed through the application of ROS scavengers.

### 3.6 Intracellular DNA/RNA damage induced by β-lactam antibiotics

During oxidative stress, ROS are known to generate distinctive oxidative damage biomarkers by targeting nucleic acid molecules. Among these biomarkers, 8-OHdG and 8-OHG emerge as prominent oxidative byproducts formed when hydroxyl radicals and superoxide anions interact with the guanine base’s eighth carbon atom in DNA and RNA. As established markers of oxidative damage, these compounds effectively reflect the oxidative stress levels experienced by cells.

**FIGURE 6:**
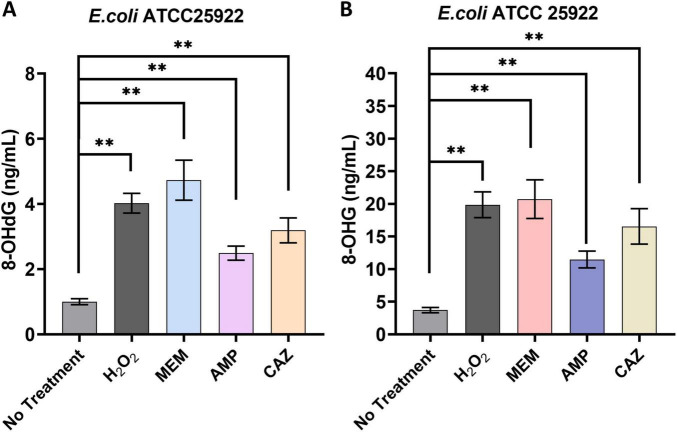
Intracellular DNA/RNA damage induced by β-lactam antibiotics. **(A)** Impact of antibacterial agents on the intracellular levels of 8-OHdG. **(B)** Impact of antibacterial agents on the intracellular levels of 8-OHG. **p < 0.01.

In our study, we investigated the effects of three distinct classes of β-lactam antibiotics-MEM, AMP, and CAZ-on the intracellular concentrations of 8-OHdG and 8-OHG in *E. coli*. Our findings indicated that all antibiotic treatment groups demonstrated a notable increase in 8-OHdG levels when compared to untreated controls. Particularly, the group treated with meropenem exhibited the most pronounced elevation, reaching 4.73 ± 0.56-fold of the levels observed in the untreated control. In contrast, hydrogen peroxide, AMP, and CAZ treatment groups displayed 8-OHdG levels of 4.02 ± 0.27, 2.49 ± 0.19, and 3.19 ± 0.34-fold, respectively (Figure 6A).

A similar trend was evident in the analysis of 8-OHG levels. All antibiotic treatment groups showed a significant rise in 8-OHG levels relative to untreated controls, with the MEM group again demonstrating the highest increase, reaching 5.57 ± 0.72-fold of the untreated control levels. The hydrogen peroxide, AMP, and CAZ groups recorded elevations of 5.34 ± 0.48, 3.08 ± 0.31, and 4.45 ± 0.66-fold, respectively (Figure 6B). These results highlight that while various β-lactam antibiotics effectively inhibit bacterial growth, they concurrently enhance oxidative damage to bacterial DNA and RNA. This observation aligns with our previous findings that antibiotics promote elevated ROS levels in bacteria. The increase in ROS levels may not only impair bacterial metabolism but also intensify their detrimental effects through further oxidative attacks on nucleic acids.

## 4 Discussion

### 4.1 Selection of concentrations that perturb bacterial metabolism via β-lactam antibiotics

The investigation into the effects of β-lactam antibiotics-MEM, AMP, and CAZ-on *E. coli* provided critical insights into establishing non-lytic concentrations for assessing antibiotic-induced bacterial death. The detection of no growth inhibition at concentrations of 0.5 μg/mL for MEM, 3.5 μg/mL for AMP, and 1.5 μg/mL for CAZ indicates that these dosages are appropriate for studying the antibiotics’ effects without introducing metabolic confounding factors. This approach aligns with recent findings indicating that non-lytic concentrations are essential for accurately determining the bactericidal and bacteriostatic properties of antibiotics while preserving cellular integrity (Kohanski et al., 2007). SEM analyses further corroborated the non-toxic nature of these antibiotic concentrations, revealing no significant alterations in cell morphology or membrane architecture in *E. coli*. Such observations are of utmost significance, as alterations in cellular structure often serve as indicators of cellular distress or demise, potentially distorting the interpretation of experimental findings. The stability of the bacterial cell architecture at these concentrations allows for a more focused investigation into the specific mechanisms through which β-lactam antibiotics disrupt bacterial metabolism. Our findings emphasize the significance of methodologically sound experimental design in antimicrobial research. Establishing appropriate antibiotic dosages facilitates a clearer understanding of the dynamics between bacterial pathogens and antimicrobial agents, paving the way for future studies aimed at elucidating the intricate interactions that govern antibiotic efficacy and resistance. These results contribute to the broader narrative of antibiotic research, indicating the need for continued exploration of antibiotic mechanisms at precise dosages that reflect *in vivo* conditions.

### 4.2 Differential metabolic profile mediated by β-lactam antibiotics

The metabolic perturbations induced in *E. coli* by distinct classes of β-lactam antibiotics reveal both shared and unique responses in bacterial metabolism. Our comprehensive analysis, leveraging UHPLC-Q-TOF/MS technology, underscores the intricate interplay of activated metabolic pathways under antibiotic stress, providing valuable insights into bacterial survival strategies. A notable commonality observed among the treatments is the pronounced impact on energy metabolism. Specifically, all three classes of antibiotics induced a significant downregulation of crucial energy metabolites such as AMP, ADP, and NADP, which can impede bacterial energy production capacity and hinder essential biosynthetic pathways (Su et al., 2018). This reduction likely compromises the cell’s ability to maintain homeostasis and respond effectively to environmental stressors, thereby supporting previous findings that depletion of energy can lead to decreased viability of bacterial cells under antibiotic pressure (Lobritz et al., 2015). The upregulation of metabolites associated with amino acid synthesis and cofactors appears to be a unique defensive response elicited by each antibiotic. For example, treatment with MEM resulted in increased levels of several amino acids (e.g., proline, leucine), potentially reinforcing cellular stress responses and mitigating the detrimental effects of ROS that are exacerbated during antibiotic exposure. Similarly, the elevation of specific cofactors such as niacin and acetyl-CoA across all treatments indicates an effort to enhance metabolic flux compensatory for inhibited pathways (Baek et al., 2011).

An intriguing aspect of the ROS response was observed, particularly in relation to downregulated antioxidant metabolites, such as glutathione, across all treatments. The decrease in both reduced and oxidized forms of glutathione suggests that antibiotic-induced oxidative stress may overpower bacterial antioxidant defenses, potentially increasing susceptibility to oxidative damage (Belenky et al., 2015). Nevertheless, the selective upregulation of metabolites associated with cellular repair indicates a potential adaptive response aimed at counteracting the oxidative effects. This highlights the dual role of metabolic shifts in promoting survival and triggering cell death pathways under antibiotic stress.

The distinct reactivity of *E. coli* to each antibiotic is noteworthy. While MEM treatment primarily promotes the synthesis of coenzymes associated with energy metabolism and amino acid metabolism, AMP treatment exhibits a significant enhancement of polyamine metabolism, which plays a critical role in cell growth and proliferation under stress conditions. Furthermore, CAZ treatment uniquely influences lipid metabolism and cell wall synthesis, suggesting a specific targeting of structural components crucial for bacterial integrity. These differential metabolic responses shed light on the unique mechanisms through which various β-lactam classes exert their antibacterial effects and can provide insights for future therapeutic strategies aimed at overcoming antibiotic resistance (Lee et al., 2018). The effects of three β-lactam antibiotics on *E.coli* metabolism also exhibit certain commonalities. All treatments significantly decreased the levels of core energy metabolites, including AMP, ADP, and NADP, thereby potentially compromising bacterial energy production capacity and impacting biosynthetic pathways. This finding suggests that antibiotic pressure detrimentally affects bacteria’s ability to maintain internal homeostasis and adapt to environmental stressors. Furthermore, all treatments resulted in the downregulation of antioxidant metabolites such as glutathione, indicating that antibiotic-induced oxidative stress may surpass the bacteria’s antioxidant defense capability. Metabolic responses to β-lactam antibiotics may exhibit variations across different bacterial strains or species, suggesting that the generalizability of our findings might be limited. Therefore, future investigations should encompass a broader spectrum of bacterial models, particularly clinical isolates with diverse resistance profiles, in order to comprehensively evaluate the applicability of our results and elucidate the metabolic dynamics within distinct bacterial contexts.

### 4.3 Metabolic reprogramming of bacteria induced by β-lactam antibiotics

The metabolic alterations observed in *E. coli* following treatment with different β-lactam antibiotics provide critical insights into the adaptive metabolic reprogramming mechanisms employed by bacteria under antibiotic stress. KEGG pathway enrichment analysis of differentially regulated metabolites revealed distinct yet overlapping metabolic pathways activated in response to each antibiotic treatment, underscoring the multifaceted nature of bacterial responses to environmental challenges. In the MEM treatment group, significant enrichment in pantothenate and CoA biosynthesis, β-alanine metabolism, and butanoate metabolism pathways suggests a robust involvement in energy-related processes and cellular regulation. The upregulation of metabolites such as 4′-phosphopantetheine and pantothenate aligns with a potential requirement to enhance coenzyme levels, supporting fatty acid synthesis and energy metabolism under stress (Belenky et al., 2015; Stokes et al., 2019). However, the downregulation of coenzyme A indicates a complex interplay between precursor accumulation and metabolic flux regulation, implying a strategic resource redirection in response to MEM-induced stress. AMP treatment notably enhanced xanthine concentrations in purine metabolism, with a striking 37.3-fold increase. This elevation likely reflects a shift toward salvaging nitrogenous bases as the cell navigates antibiotic-induced stress, highlighting the importance of purine metabolism in sustaining cellular functions during adverse conditions (Harms et al., 2016). Additionally, the substantial rise in niacin levels (79.4-fold) indicates an upregulation of nicotinate and nicotinamide metabolism, which is critical in energy metabolism and cellular signaling. The reduction in other metabolites such as adenine and ADP in conjunction with the elevation of acetyl-CoA emphasizes the multifactorial regulation of metabolic pathways as *E. coli* adapts to AMP’s inhibitory effects. CAZ treatment resulted in a significant increase in hypoxanthine levels and a decrease in AMP and GMP within purine metabolism, reflecting the adaptive shift in nucleotide homeostasis. This suggests that bacterial resilience involves strategic modulation of nucleoside levels to regulate energy demands and stress responses (Nolan et al., 2023). Furthermore, the pronounced elevation of both 4′-phosphopantetheine and pantothenate under CAZ treatment highlights the crucial role of coenzyme metabolism in cellular repair and survival.

The consistent downregulation of reduced glutathione across treatments indicates a decreased antioxidant capacity, which could potentially increase vulnerability to oxidative damage (Brynildsen et al., 2013). This pattern aligns with previous research showing how antibiotic pressure can disturb the balance of redox reactions, thus leading to bacterial death (Belenky et al., 2015). The observed increase in γ-aminobutyric acid and metabolites related to arginine and proline metabolism demonstrates an adaptive response that might help alleviate cellular harm and enhance survival when exposed to oxidative stress. However, the use of a single *E. coli* strain (ATCC 25922) may restrict the generalizability of our findings. Different bacterial strains or species may exhibit varying metabolic responses to β-lactam antibiotics, which could influence the observed outcomes. To validate our findings comprehensively, future research should include a broader range of bacterial models, particularly clinical isolates with diverse resistance profiles. Such studies would enhance our understanding of the metabolic adaptations to antibiotic stress across a more representative bacterial population and could provide valuable insights for developing targeted therapeutic strategies to combat antibiotic resistance.

### 4.4 Intracellular accumulation of ROS in bacteria induced by β-lactam antibiotics

Oxidative stress has emerged as a pivotal indicator of cellular dysfunction, with the GSH/GSSG ratio serving as a widely accepted metric for evaluating oxidative damage (Kalghatgi et al., 2013). A decrease in this ratio traditionally signifies an elevation in cellular oxidative stress (Belenky et al., 2015). In this study, the investigation of metabolic disruptions in *E. coli* exposed to three β-lactam antibiotics revealed a significant reduction in the GSH/GSSG ratio, thereby indicating substantial induction of oxidative stress by these treatments. The substantial increase in intracellular levels of ROS supports these findings. Importantly, the significant rise in ROS levels observed after MEM treatment, reaching nearly 200 ± 7% of baseline, highlights a critical response of *E. coli* to this antibiotic. The observed increases with AMP (174 ± 11%) and CAZ (152 ± 7%) also suggest that these antibiotics similarly contribute to heightened oxidative stress. The inclusion of hydrogen peroxide as a positive control further validates the robustness of our assay, establishing a clear association between β-lactam treatment and ROS generation. These results are consistent with existing literature emphasizing the role of antibiotic-induced oxidative stress as a mechanism contributing to bacterial cell death and metabolic disruption (Kohanski et al., 2007). To further elucidate the relationship between elevated levels of ROS and the efficacy of antibiotics, we investigated the impact of AA, a well-known antioxidant, on ROS production. The significant reduction in ROS levels observed in the presence of AA reinforces our hypothesis that β-lactam antibiotics elevate intracellular ROS levels, thereby contributing to oxidative damage. Specifically, the notable decreases in ROS levels following treatment with MEM (201 ± 9%), AMP (148 ± 7%), and CAZ (125 ± 3%) upon application of an antioxidant provide compelling evidence for the involvement of oxidative stress in mediating the effects exerted by these antibiotics.

The correlation between ROS elevation and antibiotic action poses a dual challenge for *E. coli*; while increased ROS levels may augment the effectiveness of antibiotics, they also pose a substantial threat to cellular integrity. This paradox underscores the significance of cellular antioxidant defenses in modulating antibiotic susceptibility and their potential involvement in the development of resistance mechanisms (Carvajal-Garcia et al., 2023). The ability of *E. coli* to counteract ROS through diverse metabolic pathways reinforces the notion that bacterial adaptability is crucial within the context of antibiotic therapy, thereby drawing attention to potential strategies for enhancing treatment efficacy through targeted utilization of antioxidants or approaches aimed at modulating redox states within bacterial populations.

Decoding the metabolic disruptions induced by β-lactam antibiotics offers crucial insights for advancing therapeutic strategies. By comprehending bacterial metabolic adaptations to antibiotic stress, more targeted treatment approaches can be developed. The potential combination of metabolic pathway inhibitors with β-lactams could effectively disrupt bacterial survival mechanisms, thereby enhancing antibiotic efficacy and reducing resistance development. This approach enables precise intervention in bacterial metabolic networks, targeting alternative energy production and stress response pathways. Such nuanced comprehension facilitates the design of combination therapies that not only improve treatment outcomes but also mitigate the risk of emerging antibiotic-resistant strains, representing a promising direction in infectious disease management. Our work proposes potential strategies for developing next-generation antibiotics that exploit metabolic vulnerabilities, potentially overcoming current resistance mechanisms. The ability to systematically design treatments targeting multiple metabolic levels represents a significant advancement in our approach to combating bacterial infections, offering promising prospects for more effective and precise clinical interventions.

### 4.5 Intracellular DNA/RNA damage induced by β-lactam antibiotics

Our study also explores the interplay between β-lactam antibiotics and oxidative stress in *E. coli*, specifically examining the formation of oxidative DNA and RNA damage biomarkers 8-OHdG and 8-OHG. These biomarkers serve as critical indicators of oxidative damage, as they are formed through the interaction of ROS with the guanine base, reflecting the extent of oxidative stress imposed upon bacterial cells (Belenky et al., 2015). The robust oxidative response is highlighted by the significant increase in 8-OHdG levels observed across all antibiotic treatment groups in this study. Notably, treatment with MEM resulted in a pronounced elevation of 4.73 ± 0.56-fold compared to untreated controls. Furthermore, the elevated levels of 8-OHdG detected in the hydrogen peroxide control group (4.02 ± 0.27-fold) provide additional evidence that hydroxyl radicals, typically generated under conditions of oxidative stress, can induce modifications in DNA and ultimately impair its function.

The consistent trend observed in 8-OHG levels enhances our comprehension of oxidative damage, with MEM once again exhibiting the highest increase at 5.57 ± 0.72-fold. The rise in 8-OHG levels holds particular significance as it signifies RNA oxidative damage, which plays pivotal roles in protein synthesis and other essential cellular functions (Magierowska et al., 2022). Understanding the implications of RNA oxidative damage is crucial, as it has the potential to disrupt translational fidelity and impact the overall metabolic state of bacterial cells. We found that although β-lactam antibiotics, such as MEM, AMP, and CAZ, exhibit potent antibacterial properties, they concurrently induce significant oxidative damage to bacterial DNA and RNA. This dual mechanism implies a potentially detrimental feedback loop in which the elevation of ROS not only contributes to the antibiotic’s efficacy in inhibiting bacterial growth but also exacerbates cellular dysfunction by inflicting oxidative damage on genetic material. Consequently, the bacteria’s ability to maintain metabolic and homeostatic functions becomes compromised, aligning with previous findings indicating increased susceptibility to additional oxidative stress (Belenky et al., 2015). Moreover, the notable and diverse elevations in 8-OHdG and 8-OHG levels detected across various antibiotics imply that individual β-lactams could potentially regulate oxidative stress via unique pathways. The discrepancies in the extent of oxidative damage might be linked to the specific mechanism of action and biological impacts exerted by each antibiotic, thereby highlighting an avenue worthy of further investigation toward comprehending bacterial response mechanisms during instances characterized by oxidative stress.

## 5 Conclusion

Our study highlights the profound metabolic perturbations induced by β-lactam antibiotics in *E.coli*, elucidating distinct yet overlapping effects across different antibiotic classes. The significant downregulation of essential energy metabolites such as AMP, ADP, and NADP indicates compromised energy metabolism, while the attenuation of antioxidant metabolites like glutathione suggests that the oxidative stress generated by these antibiotics may overwhelm bacterial antioxidant defense mechanisms. Nevertheless, the selective upregulation of metabolites associated with cellular repair processes reflects an adaptive response aimed at mitigating oxidative damage. These insights not only enhance our understanding of the metabolic consequences of bactericidal antibiotics but also underscore the intricate relationship between antibiotic action, reactive oxygen species production, and bacterial resilience; thus paving the way for future strategies to improve therapeutic outcomes against resistant bacterial strains.

## Data Availability

The raw data supporting the conclusions of this article will be made available by the authors, without undue reservation.

## Footnotes

1
http://www.ecmdb.ca
