# Supplementary material for: β-lactam antibiotics induce metabolic perturbations linked to ROS generation leads to bacterial impairment
Source: Front Microbiol. 2024 Dec 6;15:1514825. doi: 10.3389/fmicb.2024.1514825 (PMC11659197; doi:10.3389/fmicb.2024.1514825)
Supplement: Supplementary file 1 [file Data_Sheet_1.pdf]

Supplemental Information for

β-Lactam Antibiotics Induce Metabolic Perturbations Linked to ROS Generation Leads to Bacterial Impairment

Dongyang Ye[1,2,3*†], Jing Sun[1,4†], Ran Jiang[1], Jiashen Chang[1], Yiming Liu[1], Xiangzheng Wu[1], Luqi Li[5], Yihan Luo[1], Juan Wang[1,2,3], Kangkang Guo[1,2,4*], Zengqi Yang[1,2,3*]

[1]College of Veterinary Medicine, Northwest A&F University, Yangling 712100, Shaanxi, China

[2]Key Laboratory of Ruminant Disease Prevention and Control (West), Ministry of Agriculture and Rural Affairs, Yangling 712100, Shaanxi, China

[3]Key Laboratory of Animal-Derived Bacterial Resistance Monitoring (Co-Construction), Ministry of Agriculture and Rural Affairs, Yangling 712100, Shaanxi, China

[4]Experimental Animal Center, Northwest A&F University, Yangling 712100, Shaanxi, China

[5]Life Science Research Core Services, Northwest A&F University, Yangling 712100, Shaanxi, China.

Table S1 Metabolic perturbations of *E. coli* treated with meropenem

Compound	Adducts	Formula	Compound ID	Anova (*P*-value)	Fold Change
**Upregulated**					
sn-Glycero-3-phosphoethanolamine	M-H	$C_5H_{14}NO_6P$	C01233	0.000217765	55.82
Palmitoleic acid	M+H	$C_{16}H_{30}O_2$	C08362	4.7974E-06	43.99
Anthranilic acid	M+H	$C_7H_7NO_2$	C00108	3.28072E-06	35.60
Xanthine	M-H	$C_5H_4N_4O_2$	C00385	0.000163679	27.25
1-ethyladenine	M+H	$C_7H_9N_5$		3.37904E-05	26.58
Hypoxanthine	M+H	$C_5H_4N_4O$	C00262	3.10649E-06	18.34
Pterin	M-H	$C_6H_5N_5O$	C00715	1.39312E-05	12.22
Alanine	M+H	$C_3H_7NO_2$	C00041	0.00025757	8.74
γ -Aminobutyric acid	M+H	$C_4H_9NO_2$	C00334	7.81609E-05	7.94
Proline	M+H	$C_5H_9NO_2$	C00148	0.000169869	6.25
Acetyl-CoA	M+H	$C_{23}H_{38}N_7O_{17}P_3S$	C00024	0.00117095	6.21
Pantetheine	M+H	$C_{11}H_{22}N_2O_4S$	C00831	8.01448E-09	4.87
Dihydrouracil	M+H	$C_4H_6N_2O_2$	C00429	2.76462E-06	4.75
N-acetyl putrescine	M+H	$C_6H_{14}N_2O$	C02714	3.60007E-05	4.58
Cinnamic acid	M+H	$C_9H_8O_2$	C10438	5.34946E-05	4.23
Palmitic Acid	M-H	$C_{16}H_{32}O_2$	C00249	2.94643E-06	3.91
Leucine	M+H	$C_6H_{13}NO_2$	C00123	1.69492E-08	3.86
3-hydroxycinnamic acid	M+H	$C_9H_8O_3$	C00811	6.86948E-07	3.82
Pantetheine 4'-phosphate	M+H	$C_{11}H_{23}N_2O_7PS$	C01134	1.73614E-06	2.98
Inosine	M+H	$C_{10}H_{12}N_4O_5$	C00294	0.000169796	2.29
Uracil	M+H	$C_4H_4N_2O_2$	C00106	0.000617408	2.16

N1-Acetylspermidine	M+H	$C_9H_{21}N_3O$	C00612	0.00306904	2.15
Maltotetraose	M+H	$C_{24}H_{42}O_{21}$	C02052	0.00473059	1.94
**Downregulated**					
Adenine	M+H	$C_5H_5N_5$	C00147	0.000276708	-1.85
Adenosine	M+H	$C_{10}H_{13}N_5O_4$	C00212	0.000170564	-2.00
Fuculose 1-phosphate	M-H	$C_6H_{13}O_8P$	C01099	0.000673723	-2.54
Allose	M-H	$C_6H_{12}O_6$	C01487	3.25963E-05	-2.79
Oxidized glutathione	M+H	$C_{20}H_{32}N_6O_{12}S_2$	C00127	5.20662E-08	-3.97
Cytidine	M+H	$C_9H_{13}N_3O_5$	C00475	0.000169666	-3.97
UDP-N-acetyl-D-mannosamine	M-H	$C_{17}H_{27}N_3O_{17}P_2$	C01170	0.000827443	-4.06
Reduced glutathione	M-H	$C_{10}H_{17}N_3O_6S$	C00051	0.000184751	-4.19
UDP-N-acetylmuramate	M-H	$C_{20}H_{31}N_3O_{19}P_2$	C01050	0.000896465	-5.80
Dephospho-CoA	M+H	$C_{21}H_{35}N_7O_{13}P_2S$	C00882	0.000413399	-5.87
AMP	M+H	$C_{10}H_{14}N_5O_7P$	C00020	0.000402801	-5.98
UMP	M+H	$C_9H_{13}N_2O_9P$	C00105	0.00077535	-6.73
ADP	M+H	$C_{10}H_{15}N_5O_{10}P_2$	C00008	7.43504E-05	-7.33
NADP	M-H	$C_{21}H_{28}N_7O_{17}P_3$	C00006	0.000397117	-8.63
ADPribose	M+H	$C_{15}H_{23}N_5O_{14}P_2$	C06743	2.92411E-05	-9.00
NAD	M+H	$C_{21}H_{27}N_7O_{14}P_2$	C00003	0.000192767	-13.13
Ornithine	M-H	$C_5H_{12}N_2O_2$	C01602	0.000338928	-13.16

Table S2 Metabolic perturbations of *E. coli* treated with ampicillin

Compound	Adducts	Formula	Compound ID	Anova (*P*-value)	Fold Change
**Upregulated**					
CDP-1,2-dihexadec-9-enoylglycerol	M+H	$C_{44}H_{77}N_3O_{15}P_2$		4.38217E-07	87.05
Niacin	M+H	$C_6H_5NO_2$	C00253	0.000129097	79.41
FMN	M+H	$C_{17}H_{21}N_4O_9P$	C00061	7.94107E-07	54.23
Xanthine	M+H	$C_5H_4N_4O_2$	C00385	1.37679E-06	37.30
2-Amino-2-butenoic acid	M+H	$C_4H_7NO_2$	C01771	0.00017393	23.24
4-Aminobenzoic acid	M+H	$C_7H_7NO_2$	C00568	3.31173E-06	15.64
Hypoxanthine	M+H	$C_5H_4N_4O$	C00262	1.2389E-06	11.04
Acetyl-CoA	M+H	$C_{23}H_{38}N_7O_{17}P_3S$	C00024	5.43393E-05	9.98
Nicotinamide	M+H	$C_6H_6N_2O$	C00153	9.93599E-06	8.86
Methionine	M+H	$C_5H_{11}NO_2S$	C00073	2.84827E-06	4.00
Phenylacetaldehyde	M+H	C_8H_8O	C00601	0.000396147	1.92
α -Linolenic acid	M+H	$C_{18}H_{30}O_2$	C06427	0.000138636	1.90
Leucine	M+H	$C_6H_{13}NO_2$	C00123	8.825E-05	1.89
Taurocholic acid	M+H	$C_{26}H_{45}NO_7S$	C05122	0.000873467	1.67
N-acetyl putrescine	M+H	$C_6H_{14}N_2O$	C02714	0.000810165	1.78
γ -L-Glutamyl-L-cysteine	M+H	$C_8H_{14}N_2O_5S$	C00669	0.00014796	1.86
5-Methylthioadenosine	M+H	$C_{11}H_{15}N_5O_3S$	C00170	1.37092E-05	2.20
**Downregulated**					
Dephospho-CoA	M-H	$C_{21}H_{35}N_7O_{13}P_2S$	C00882	0.000724958	-1.70
Tyrosine	M+H	$C_9H_{11}NO_3$	C00082	0.002748118	-1.90
Reduced glutathione	M-H	$C_{10}H_{17}N_3O_6S$	C00051	0.000324904	-2.72

Fructose 1-phosphate	M-H	$C_6H_{13}O_9P$	C01094	0.000131597	-3.00
Arginine	M+H	$C_6H_{14}N_4O_2$	C00062	0.001206301	-3.14
AMP	M+H	$C_{10}H_{14}N_5O_7P$	C00020	0.002636698	-3.66
ADP	M-H	$C_{10}H_{15}N_5O_{10}P_2$	C00008	0.00085378	-3.66
Allose	M-H	$C_6H_{12}O_6$	C01487	1.54074E-05	-3.67
UDP	M-H	$C_9H_{14}N_2O_{12}P_2$	C00015	6.15525E-06	-3.72
Palmitic Acid	M-H	$C_{16}H_{32}O_2$	C00249	2.93498E-06	-9.77
Adenine	M+H	$C_5H_5N_5$	C00147	0.000140363	-12.57
UDP-N-acetyl-D-mannosamine	M-H	$C_{17}H_{27}N_3O_{17}P_2$	C01170	0.000159803	-14.65
Pantothenate	M+H	$C_9H_{17}NO_5$	C00864	4.0059E-05	-56.96
12-Ketodeoxycholic acid	M+H	$C_{24}H_{38}O_4$		0.000339489	-85.23
2,3-Dihydro-2,3-dihydroxybenzoate	M+H	$C_7H_7O_4$	C04171	0.000134474	-87.76

Table S3 Metabolic perturbations of *E. coli* treated with ceftazidime

Compound	Adducts	Formula	Compound ID	Anova (*P*-value)	Fold Change
**Upregulated**					
2-Amino-2-butenoic acid	M+H	$C_4H_7NO_2$		1.11114E-05	42.27
L-Ala-γ-D-Glu-Meso-Diaminopimelic acid	M+H	$C_{15}H_{26}N_4O_8$		3.88856E-08	50.13
Niacin	M+H	$C_6H_5NO_2$	C00253	4.26855E-05	43.25
Pantothenate	M+H	$C_9H_{17}NO_5$	C00864	1.92342E-07	55.83
Cytidine	M+H	$C_9H_{13}N_3O_5$	C00475	1.09641E-05	44.52
Dihydroneopterin	M+H	$C_9H_{13}N_5O_4$	C04874	5.39988E-05	31.22
N2-Succinyl-L-arginine	M+H	$C_{10}H_{18}N_4O_5$	C03296	0.000115433	30.45
Pantetheine 4'-phosphate	M+H	$C_{11}H_{23}N_2O_7PS$	C01134	3.10417E-09	26.21
Hypoxanthine	M+H	$C_5H_4N_4O$	C00262	3.18453E-06	18.54
Carnitine	M+H	$C_7H_{15}NO_3$	C00318	6.02062E-06	14.87
γ-butyrobetaine	M+H	$C_7H_{15}NO_2$	C01181	6.68103E-05	12.26
Palmitoleic acid	M+H	$C_{16}H_{30}O_2$	C08362	3.55323E-08	10.03
Proline	M+H	$C_5H_9NO_2$	C00148	3.03719E-12	9.07
Guanosine	M+H	$C_{10}H_{13}N_5O_5$	C00387	4.38783E-05	2.99
FMN	M+H	$C_{17}H_{21}N_4O_9P$	C00061	4.53319E-08	2.71
**Downregulated**					
Uracil	M-H	$C_4H_4N_2O_2$	C00106	1.71227E-05	-4.92
Methyl bisulfate	M-H	CH_4O_4S		0.000488963	-1.92
Acetyl-maltose	M-H	$C_{14}H_{24}O_{12}$	C02130	0.000426934	-1.87
Adenine	M+H	$C_5H_5N_5$	C00147	0.004599519	-2.39
Myristic acid	M-H	$C_{14}H_{28}O_2$	C06424	4.57794E-05	-2.53

AMP	M+H	$C_{10}H_{14}N_5O_7P$	C00020	0.002645873	-2.64
Maltopentaose	M-H	$C_{30}H_{52}O_{26}$		0.000283939	-2.64
Riboflavin	M-H	$C_{17}H_{20}N_4O_6$	C00255	0.000217531	-2.73
Tartaric acid	M-H	$C_4H_6O_6$	C00898	0.00186735	-2.77
Pantoate	M-H	$C_6H_{11}O_4$	C00522	0.001798093	-2.88
Maltose 6'-phosphate	M-H	$C_{12}H_{23}O_{14}P$	C02995	0.000123386	-2.98
Deoxyinosine	M-H	$C_{10}H_{12}N_4O_4$	C05512	0.000880952	-3.09
Trehalose 6-phosphate	M-H	$C_{12}H_{23}O_{14}P$	C00689	0.000482348	-3.40
Fuculose 1-phosphate	M-H	$C_6H_{13}O_8P$	C01099	0.000598036	-4.27
1-deoxy-D-xylulose 5-phosphate	M-H	$C_5H_9O_7P$	C11437	9.55347E-05	-4.37
GMP	M-H	$C_{10}H_{14}N_5O_8P$	C00144	7.19543E-05	-4.47
Lysine	M-H	$C_6H_{14}N_2O_2$	C00047	0.000194425	-4.56
Glucosamine	M-H	$C_6H_{13}NO_5$	C00329	0.000208519	-4.68
2,3-Dihydro-2,3-dihydroxybenzoate	M-H	$C_7H_7O_4$		4.76084E-05	-4.84
Fructose 1-phosphate	M-H	$C_6H_{13}O_9P$	C01094	2.89446E-05	-5.72
Cys-Gly	M-H	$C_5H_{10}N_2O_3S$	C01419	0.000417896	-7.09
Reduced glutathione	M-H	$C_{10}H_{17}N_3O_6S$	C00051	9.1278E-05	-7.45
D-Alanyl-D-alanine	M+H	$C_6H_{12}N_2O_3$	C00993	0.001106488	-8.13
N1-Acetylspermidine	M+H	$C_9H_{21}N_3O$	C00612	0.000563053	-8.52
Dephospho-CoA	M-H	$C_{21}H_{35}N_7O_{13}P_2S$	C00882	5.42961E-05	-8.81
NADP	M-H	$C_{21}H_{28}N_7O_{17}P_3$	C00006	0.000394459	-9.11
Ornithine	M-H	$C_5H_{12}N_2O_2$	C01602	0.00026595	-9.24
UDP-N-acetyl-D-mannosamine	M-H	$C_{17}H_{27}N_3O_{17}P_2$	C01170	0.000193093	-11.84
